# Development of a Conceptual Model of Occupational Stress for Athletic Directors in Sport Contexts

**DOI:** 10.3390/ijerph19010516

**Published:** 2022-01-04

**Authors:** Ye Hoon Lee, Hyungsook Kim, Yonghyun Park

**Affiliations:** 1 Division of Global Sport Industry, Hankuk University of Foreign Studies, Seoul 17035, Korea; leeye22@o365.hufs.ac.kr; 2 Department of Cognitive Sciences, School of Intelligence, Hanyang University, Seoul 04763, Korea; 3 Graduate School of Public Policy, Hanyang University, Seoul 04763, Korea; 4 HY Digital Healthcare Center, Hanyang University, Seoul 04763, Korea; yhpark81@hanyang.ac.kr

**Keywords:** athletic director, job stress, organizational behavior, occupational stress model

## Abstract

Previous studies have reported that occupational stress is a determinant risk factor for both chronic diseases and job performance among organizational leaders. Every occupation has its own culture and occupational climate influencing organizations within the industries. Thus, due to the idiosyncratic features inherent in sports, athletic directors may experience different occupational stressors. To date, there has been no comprehensive review of the occupational stress in athletic director contexts. Thus, based on the literature on both occupational stress and sport leadership, this study proposes a conceptual framework of occupational stress in sport leadership. The model identifies the five higher-order themes of occupational stressors and their associations with the first-level outcomes of individuals and the second-level outcomes of organizations. It also includes the two higher-order moderators of personal and organizational factors. It is hoped that this initiative can invoke interest in this topic to provide health-enhancing environments for athletic directors and quality sport services to society.

## 1. Introduction

Leadership position is a rewarding but stressful occupation with a wide variety of threats and challenges from both inside and outside organizations [1,2]. The Center for Creative Leadership reported that 88% of leaders mentioned substantial stress in their work situations [3]. Although a certain level of stress can lead to positive work outcomes (i.e., increased motivation and concentration), repetitive and chronic stress has been found to negatively affect not only leaders’ physical and mental health but also decision-making process, adaptive leadership behaviors, and leader–member relationships [4,5]. In turn, these negative consequences were found to determine organizational performance through increasing stress and burnout in followers as a leader’s stress could be transferred to the mood states in followers (see [6]).

As sport leaders, athletic directors experience the same phenomenon [7,8]. The trend in school athletics has led to the growth of the responsibilities for the conduct of athletic programs because schools view athletic directors as a part of the school [9]. Subsequently, athletic directors have various tasks (e.g., performance-related, administrative, and public duties) that may cause stress [10,11], and they are at risk of burning out [7]. Considering the detrimental effects of occupational stress in general, it is critical to explore the factors creating such stress in academic directors in advance to prevent health problems and decrease leadership effectiveness.

When we consider the unique power of sports in society, the importance of investigating occupational stress in athletic directors becomes even greater [12], as they can indirectly influence young athletes through influencing athletic coaches. For example, by promoting a common value system that develops young athletes’ character and well-being and ensuring coaches adhere to it, athletic directors can play a critical role in overcoming the mindless emphasis on the winning-at-all-costs attitude surrounding interscholastic sports and fostering young student–athletes’ well-being and development. However, research has revealed that leaders under stress are likely to become self-focused and less likely to assume a team perspective [13], and in turn, they act in destructive ways toward their followers through abusive leadership behavior [4]. Under stress, it is possible that athletic directors may lose sight of young athletes’ development and other long-term planning for programs, facilities, and budgets in their quest for a short-term visible goal of winning [14]. To prevent this scenario in advance, it is important to understand the process of athletic directors’ stress. However, sport science scholars have largely ignored this topic among athletic directors. In fact, most stress research in this area has been conducted in the field of sport psychology with a focus on coaches and has investigated its impact on individual outcomes such as burnout, motivations, and athletic performance [15,16].

Taken together, to generate further interest in this topic, this study conducted a comprehensive review on occupational stress among athletic directors. The primary purpose was to explain the factors that that may be associated with athletic directors’ occupational stress and discuss how this concept can be incorporated into a sport leadership context and used to predict individual and organizational outcomes. Ultimately, it is hoped that this initiative can provoke interest in this topic to provide health-enhancing environments for sport leaders and quality sport services to society. To attain this goal, several existing models in other domains including Karasek’s (1979) job demand–control (JDC) model, Siegrist’s (1996) effort–reward imbalance (ERI) model, and a person–environment (P-E) fit model [17] were critically reviewed. Based on the review, the possible application in a sport context was discussed via the development of a conceptual model of occupational stress among athletic directors.

## 2. Conceptual Model of Occupational Stress in Sport Leadership

**Figure 1:**
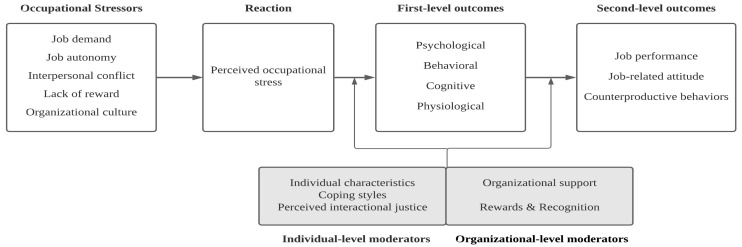
Overall conceptual model of occupational stress for sport leaders.

The model identified the five different sources of occupational stress (see Table 1), leading to stress experience, the first-level outcomes of individuals, and the second-level outcomes of organizations. Furthermore, the model describes several moderators in the association between stress experience and the first-level outcomes and between the first- and the second-level outcomes (see Figure 1).

### 2.1. Definition of Occupational Stress

Historically, the primary motivation to study the topic of stress was to determine the cause of human disease and the solutions to prevent it. Stress refers to all types of disturbances that cause discomfort or disturbance in everyday life, such as various stimuli from the external environment, those that occur physiologically, and mental conflict [18]. Stress can be divided into eustress and distress. Eustress refers to a reaction that generates a sense of excitement and joy and that produces rewards for achieving goals; it is a desirable strain that imparts a sense of help and happiness to life. Conversely, distress is an uncomfortable or harmful state generated by unpredictable or uncontrollable situations. This negatively affects mental and physical functions, which can lead to illness or feelings of helplessness [19]. To prevent its negative consequences, a substantial amount of research has been conducted on stress in the fields that study the human body and mind, such as medicine, psychology, genetics, molecular biology, neuroscience, and immunology [20].

Extending the concept of stress, occupational stress refers to negative physical and psychological reactions that occur in the workplace or within the organization; these reactions are generated by the state of incongruity between the individual’s ability or desire and job-related tasks or environmental factors [20]. Regarding the causes, the National Institute of Occupational Safety and Health [21] stated that job conditions are the primary cause of occupational stress. Gurung (2006) suggested nine causes of occupational stress, including excessive job burden, role conflict, role ambiguity, discrimination, failure to receive promotion due to prejudice, inadequate social networks, lack of job control, role multiplicity, and lack of challenging tendencies.

### 2.2. Sources of Occupational Stress

#### 2.2.1. Job Demands

The JDC model identified the first higher-order stressor of job demands that refers to the degree of perceived burden from the job itself. Stress occurs when the job requirements are beyond the employees’ capabilities or when the individual lacks the ability to perform the job required [22]. More specifically, this model identified the three lower-order stressors: general job demands that can be applied to all domains, administration-related, and competition-related job demands specific to sport leadership contexts.

##### Administration-Related Job Demands

One of the notable differences between athletic directors and coaches as leaders is that the former work largely in an office instead of on the field or court. Office duties usually comprise paper- or computer-based tasks necessary for the settlement of deadlines [23]. These may be inevitable duties for athletic directors as they relate to the legal aspects of the jobs and the particular needs of athletes. Thus, tight deadlines with time constraints can be one occupational stressor among athletic directors. 

Additionally, researchers [21] notes that heavy workloads are one of the top stressors in the workplace. Among athletic directors, the problems related to job demands are characterized as “all day and after dark” work, saddled with new duties and extra responsibilities, including operating school-sponsored tournaments and workshops; managing medical supplies and related equipment; constructing, maintaining, and repairing of physical facilities; and paying coaching salaries and transportation costs [9,10]. It also includes athlete disciplinary issues and issues regarding team performance.

In addition to these formal responsibilities, the business aspect of athletic programs has grown so large that athletic directors must develop marketing and promotional strategies based on skills and knowledge associated with business administration. High school and college athletics are facing enormous budgetary pressure as rising costs of operations and educational budget cuts strain athletic budgets and often require large expenditures of money [24]. Thus, athletic directors may experience pressure from selling relevant merchandises that may be related to the apparent primary focus of the sports [25]. They have also begun to face new challenges, necessitating creating new and unique ways to generate funds to ensure their sport programs are financially secure [23].

Further, competition among institutions for public exposure and student–athletes places great demands on athletic directors as they can be viewed as ambassadors of schools [9]. With every action and outcome under such severe scrutiny, athletic directors need to consider how they present their emotions and true feelings in public, which can provide additional demands [26]. Furthermore, constant “talk radio,” anonymous instant experts, and parents on social media can easily voice their opinions about the athletic programs that often conflict with the goals of athletics and highlight the athletic directors’ mistakes [27].

Athletic directors are expected to handle and manage numerous policy areas including eligibility, gender equity, funding, and amateurism [23]. Promoting academic eligibility for all student–athletes and ensuring they abide by such regulations may be one of the most important duties. Thus, athletic directors’ duties include communicating such expectations to students and parents to make them aware of the additional requirements [23]. Additionally, despite its important implications, complying with Title IX legislation also put strains on athletic directors as numerous lawsuits have been initiated by athletes and parents that challenge actual Title IX compliance [26,28].

**Proposition** **1.** *Athletic directors will experience occupational stress from the general job demands including time pressure, workload, requirements for business techniques, budgetary pressure, policy compliance, and public relations*.

##### Competition-Related Job Demands

In the case of athletic directors, job demands related to competition can be particularly burdensome. A school’s athletic program is a window into its reputation, and winning takes priority over fun, participation, and life lessons. Subsequently, athletic directors may feel pressure inside and outside schools [10]. To ensure better team performance, it is necessary to hire qualified coaches. While hiring qualified coaches can be one of the most significant stressors among athletic directors, funding issues will lead to a decline in coaching stipends, which in turn makes it difficult to find suitable coaches.

Unlike business leaders, athletic directors usually perform double duties as educators and administrators, often overseeing student–athlete activities, discipline, and academic department, in addition to all the sports programs. Many athletic directors also coach sports and carry a full teaching load [9]. Although some similarities do exist, the roles of educators and administrators are not considered identical, as there are differences in terms of occupational goals and objectives, skill sets and abilities, responsibilities, and reward systems in the two occupational settings [29]. As administrators, athletic directors work as department spokesperson whenever they have contact with several people inside and outside the department. In this process, they need to share the program philosophy with the public, promote student participation, coordinate coaching activities, and evaluate the effectiveness of the program. As educators, they also need to consider young student–athletes’ educational experience. Subsequently, these unique characteristics can create role stressors such as role conflict and ambiguity, resulting in increased job demands [30].

Additionally, unique physical factors that can cause occupational stress can be viewed as the condition of external environment directly related to performance in athletics. These factors can include concerns related to sport facility constructions/renovations and training environment. Obviously, sports cannot be played without any enclosed physical facilities built, installed, or established as a location [31]. However, there have been increased demands in terms of new athletic facility constructions/renovations. Thus, it may cause stress to build and maintain state-of-art athletic facilities in sports.

**Proposition** **2.** *Athletic directors will experience occupational stress from competition-related job requirements including pressure to win, a conflict between performance and development goals, and facility issues*.

#### 2.2.2. Job Autonomy

The second higher-order stressor of occupational stress is job autonomy, which refers to the level of control one has over their work [32]. Previous organizational behavior literature demonstrated that when individuals have less control over their jobs and outcomes, they will make frequent mistakes and show psychological distress [33,34].

The main product athletic directors are managing is competitions, which are characterized by unpredictable outcomes [35,36]. In fact, athletes can contribute to the outcomes of competitions through their performance; furthermore, coaches play a critical role in this process by training and inspiring athletes. However, athletic directors’ influences on competition outcomes can be limited and indirect through hiring players/coaches or managing training/competition environments. Thus, they may experience stress from the unpredictable nature of sport competitions.

In addition to the unpredictable outcomes, there are numerous uncontrollable factors in athletics such as player/coach turnover, injuries, and athlete, coaches, and fans’ deviant behaviors. For example, after the Penn State child sex abuse scandal, the athletic director, Tim Curley, was charged with several offenses including perjury and obstruction of justice, and the Penn State Board of Trustees terminated his contracts [37]. This is a somewhat unique situation as leaders in business are less likely to be responsible for and be terminated due to the misconduct of followers. Athletes’ injuries are another unpredictable incident athletic directors must cope with. For example, growing concerns have been expressed regarding concussions, with particular emphasis at the level of high and middle school sports [38]. This can cause stress among athletic directors in their attempts to prepare and monitor such incidents.

**Proposition** **3.** *Athletic directors will experience occupational stress from a lack of job autonomy including unpredictable outcomes and uncontrollable athletic-related issues (e.g., player/coach turnover, injuries, and deviant behaviors)*.

#### 2.2.3. Interpersonal Conflict

From the P-E fit model, the current model proposes the third higher-order stressor of interpersonal conflict, which refers to “the awareness of interpersonal incompatibilities, including affective components such as the experience of tension and friction [39] (p. 23)”. The P-E fit model assumes that when individuals cannot successfully manage interpersonal conflict at work, they are more likely to experience stress reactions [17]. Previous literature has reported that episodes of interpersonal conflict could affect individual well-being [40] and other important individual outcomes such as job dissatisfaction, lower organizational commitment, and higher turnover intentions [41,42,43]. These interpersonal conflicts can be induced by athletic directors’ interaction with coaches, student–athletes, parents, and booster clubs.

##### Working with Coaches and Student-Athletes

High-profile coaches and athletes can wield a great degree of power and legitimacy in the organization, and their celebrity status can challenge athletic directors’ legitimate authority to a much greater extent than internal stakeholders in other business sectors [44,45]. The quality of the core products in sports is largely determined by high-performance practitioners (i.e., coaches and athletes). Thus, athletic directors may struggle not only with their lack of power over the product but also challenges from coaches and athletes who can exert significant influence on the core product [44,46].

Moreover, many coaches often have certain expectations about what their leaders need to provide to enable them to be successful. At times, these expectations and needs may be at odds with the budgetary situations or the leader’s preferred direction for the organization (e.g., winning vs. development and player recruitment). In contrast, athletes who have traditionally lacked voice and power in some contexts (e.g., intercollegiate sport; see [47]) may gain power and influence over leaders when advocating for changes to improve their participation experiences (e.g., National Collegiate Athletic Association reforms to meal allowance and full cost of scholarships in Division I; [44]).

**Proposition** **4.** *Athletic directors will experience occupational stress from interpersonal conflict with coaches and athletes*.

##### Working with Parents and Booster Clubs

Parents can play a critical role in youth sports [48]. However, extensive evidence suggests that parents’ involvement in youth sports have become increasingly problematic [49]. For example, due to their concerns about the welfare of their children, many parents have been overly involved with their children’s athletic career, spending thousands of dollars, overemphasizing winning, showing unrealistic ideas of their children’s ability, and even criticizing them [10,50,51]. Additionally, if parents are not happy with coaches’ styles, they tend to contact athletic directors to share their concerns via email and phone calls. Thus, athletic directors are expected to cope with these overly involved parents [10], and this process can become a stressor. As the recent development of technology in media and new media allows these clients to easily access and keep track on team performances, this is particularly relevant to the sport setting [27].

Some athletic programs also have booster clubs, which are primarily comprised of parents and alumni who are interested in the success of the athletic program and who donate their time and money to help sustain the programs. Similarly, while the vast majority of boosters are involved with the athletic programs to further the interests of young athletes, this initiative also puts pressure on athletic directors because some consider return on investment in terms of either financial repayment or pride [26]. Booster clubs also support athletics only to indulge a need to influence the program or the people within it. These cases often violate the spirit and the rules governing interscholastic athletics; thus, athletic directors should do all they can to control their involvement [51].

**Proposition** **5.** *Athletic directors will experience occupational stress from interpersonal conflict with parents and booster clubs.*

#### 2.2.4. Lack of Rewards

As a source of occupational stress among athletic directors, the ERI model proposes the fourth higher-order stressor: a lack of rewards in terms of respect, recognition, monetary rewards, and the opportunity for professional development. For example, athletic directors may feel dissatisfaction from a lack of financial rewards for what they consider to be a time-consuming and stressful job. Lee (2020) reported that 27.9% of high school athletic directors had a salary range of $70,000–$89,999, while 24.4% had a range of $50,000–$69,999 depending on the type of school (e.g., public or private), while the assigned duties extend beyond running the athletic program.

Directors can also feel stress from the lack of recognition for their endeavors and successes as athletic administrators. Unfortunately, there is a generally significant misunderstanding regarding the job of athletic directors, especially at the high school level, as “some teachers regard athletic directors as former coaches who are killing time between their last game and their first day of retirement” [26] (p. vi). Subsequently, they have been treated as less important members in the school system and must cope with the marginalization of their duties and physical and intellectual isolation [26].

Finally, athletic directors may experience frustration due to a lack of opportunities for professional development. Most reach their positions as the conclusion of a career from coach [9]. This indicates that athletic directors begin their career at somewhat older ages, between 40 and 50, with sufficient athletic coaching experience and a lack of office experience. This new working condition may lead them to feel the need for obtaining management skills and knowledge. However, unless the school system or sport governing bodies provide sufficient opportunities for their educational development, there will be a significant gap between what they are expected to do and what they can actually do. This also includes new technologies and processes related to internet systems.

**Proposition** **6.** *Athletic directors will experience occupational stress due to a lack of rewards in terms of monetary rewards, recognition, and opportunities for professional development*.

#### 2.2.5. Organizational Culture

Based on the P-E fit model, the last higher-order stressor in the current model is organizational culture in sports. Welty Peachey et al. (2015) note that this conceptual model considers organizational culture as a significant factor explaining the occupational stress process in sport leadership. Previous organizational sciences literature has stated that organizational culture functions as an important stress mechanism [52], as an incongruence between individual characteristics and organizational culture can generate occupational stress [17]. One of the important topics in this section is female athletic directors’ stress experience in the specific organizational culture within sports.

Organized sport has been created, maintained, and shaped by men, and sport culture has been considered as male-dominated, -identified, and -centered. That is, ability and performance qualifications are associated with masculinity, and sports are “a man’s world” that emphasizes values associated with men and masculinity [51]. Thus, women have been underrepresented in decision-making positions in sports [53]. For example, in 1998, 19% of athletic directors in the NCAA were females; this figure remains somewhat similar at 20% in 2012, while the proportion of female athletic directors is the lowest in Division I and the highest in Division III [54]. It is no surprise that the exclusion and underrepresentation of women in administration exists worldwide [55].

Coakley [51] noted that when women achieve the position of decision-making power, they usually face challenges not only from the work but also from the male-oriented culture. Compared to men, they are less likely to feel welcome and fully included in the department, which in turn leads them to have higher rates of stress, job dissatisfaction, and turnover [51]. The female-specific stressors of athletic directors can range from the attitude and tone of male coaches, parents, and stakeholders, restrictions on promotion, and the expectation of child rearing. Another stress factor specific to female athletic directors is sexual harassment. It has been reported that some sport organizations have records of being negligent in terms of controlling sexual harassment and responding to complaints from women who wish to be taken seriously in the structure and culture of sport organizations and programs [51].

**Proposition** **7.** *Female athletic directors will experience occupational stress from the male-oriented culture inherent in sport organizations and sports*.

### 2.3. Occupational Stress and Outcomes

Previous research has noted that these occupational stressors cause various consequences including individual- and organizational-level results [56]. Employees faced with a stressor will engage in coping or avoidance reactions, and their bodies and minds will try to restore homeostasis. In this process, it is more likely that the body’s adaptive energy will be depleted and illness can occur, which can in turn affect their work performance [57,58,59].

#### 2.3.1. First-Level Outcomes

The first-level outcomes caused by occupational stress in individuals can be divided into psychological, behavioral, cognitive, and physiological responses (e.g., [60,61]). Psychological responses include depression, anxiety, frustration, emotional instability, indifference, aggression, neurasthenia, and a lack of self-esteem. For example, Fortes et al. [62] reported that occupational stress was positively associated with depression. Behavioral responses toward stress include weariness, loss of concentration, irritated reactions, smoking, excessive drinking, substance abuse, and overeating. Cognitive responses include low memory and difficulty in decision-making, acquiring new information, and concentration. Finally, physiological responses include elevation of blood sugar levels and blood pressure, mouth dryness, dyspnea, and cardiac disorders.

**Proposition** **8.** *Proposed organizational stressors will have a significant positive impact on psychological, behavioral, cognitive, and physiological responses of the first-level outcomes*.

#### 2.3.2. Second-Level Outcomes

By extension, occupational stress can be problematic at the organizational level, as negative individual outcomes can lead to reduced job satisfaction and performance (e.g., [58]), increased withdrawal and turnover [63], higher rates of accidents [64], and drug and alcohol use [65]. For example, excessive alcohol use caused by occupational stress results in a loss of productivity because it interferes with the worker’s ability to perform both quantitatively and qualitatively [65].

**Proposition** **9.** *The first-level outcomes will have significant negative impacts on the second-level outcomes of job satisfaction, job performance, withdrawal and turnover, accidents, and drug and alcohol use*.

### 2.4. Moderators

Although previous literature has typically stated that occupational stress significantly affects individual and organizational outcomes [6], notably, the strength of the associations varies depending on several moderating factors [66,67]. For example, Hengartner et al. [67] found that the influence of occupational stress on health outcomes was significantly moderated by personality. The P-E fit model identified the two higher-order moderators of personal and organizational factors that can influence the associations among the proposed constructs, as efforts to resolve stress-related problems should be made at the personal and the organizational level. This step is necessary to minimize the negative consequences of occupational stress and maximize the organizational effectiveness within athletic departments.

The first higher-order moderator includes sport leaders’ individual characteristics including Type A behavior patterns, obsessive passion, personality, esteem, previous athletic experience, age, gender, and education level. For example, obsessive passion refers to a strong desire to engage in an activity with a loss of controllability toward it, thus feeling pressured to engage in it persistently [68]. This factor has been found to be positively associated with the experience of burnout and stress in sports [35,69], as performers with a high level of obsessive passion tend to experience low implicit self-esteem [70], that is, negative situational emotions [68]. Unlike other domains, it is important to note that previous sport management literature identified a positive link between obsessive passion and work-related outcomes of affective commitment, job satisfaction, job involvement, and organizational citizenship behavior [71]. Extending this argument, sport leaders’ obsessive passion may have different moderating influences on the association between occupational stressors and first-level outcomes and that between first- and second-level outcomes. That is, obsessive passion may strengthen the detrimental effects of occupational stressors on the occupational well-being of first-level outcomes, whereas it may have a significant mitigating effect of the first-level outcomes on the second-level outcomes. Future studies may need to further investigate this paradox of obsessive passion in sport organizations.

Additionally, athletic directors’ coping styles are based on their experience in stressful events. Coping is defined as “the cognitions and behaviors, adopted by the individual following the recognition of a stress encounter, that are in some way designed to deal with that encounter or its consequences” [72] (p. 7). Thus, depending on how athletic directors cope with the stressful situations, they may experience different consequences either in a positive or negative manner [29,73]. For example, previous literature has noted that the extent to which individuals appraise future threats as challenges and opportunities for new achievements (i.e., proactive coping strategy) was found to have a negative association with stress and burnout [74]. In contrast, surface acting strategy (i.e., the process of faking outward expressions regardless of their true feelings) was found to have a positive association with stress and burnout [75].

**Proposition** **10.** *Athletic directors’ personal characteristics and coping strategies will moderate the association between occupational stressors and first-level outcomes, and that between first- and second-level outcomes.*

In the proposed model, the second higher-order moderator is the organizational-level support that sport governing bodies or institutions may provide. For example, organizational support buffers the effects of stress by moderating the stress reaction [57]. This can be done by assisting the individual with coping [76] and enhancing self-esteem and perceived control over tasks [77]. There are various kinds of organizational support such as tangible resources (e.g., salary, opportunities for professional development, and coping intervention programs) and intangible resources (e.g., recognition, esteem, and helpful information [78]). For example, schools can offer a mentoring program for advice and certification and leadership programs for professional development. These programs may cover specific topics such as how to deal with booster clubs and parents and how to maintain facilities.

Furthermore, the negative consequences of stressors and health problems may be reduced through a reasonable adjustment of the compensation system through a fair performance evaluation process, which may enhance athletic directors’ perception of distributive and procedural justice [79]. Particularly, a salary that can reflect the actual time commitment and increased responsibilities should be considered. In addition to monetary and process-based fairness, organizations should place importance on increasing athletic directors’ interactional justice, which focuses on how fairly members are treated by the organizations. Interactional justice largely consists of interpersonal fairness, which indicates the perceived fairness of the quality of treatment that the members receive in the interaction with organizations such as honesty, trust, courtesy, and respect [80]. Interactional justice was found to increase the likelihood of job performance and reduce that of counterproductive work behaviors such as absenteeism, sabotage, cyber loafing, theft, and knowledge hiding [81]. For example, employees with low levels of perceived interactional justice will be more likely to have an increased level of negative emotions and toward the organizations and reject the norms of the organizations, which can manifest in counterproductive work behaviors and reduce organizational effectiveness [82,83].

**Proposition** **11.** *Organizational support and perceived interactional justice will moderate the association between occupational stressors and first-level outcomes and that between first- and second-level outcomes.*

## 3. Conclusions and Future Research Directions

Based on the previous literature on occupational stress in other domains and sports, a conceptual model of occupational stress in athletic directors is proposed to properly reflect the features of the sport context and its culture. The model contains the five sources of occupational stress, the actual stress experience, the first-level individual outcomes, and the second-level organizational outcomes. The individual- and the organizational-level moderators that can mitigate the negative consequences of occupational stress are also identified.

The current review addresses several directions for future research in sports science. First, it is necessary to develop the psychometrically validated questionnaire specific to sport leaders for future quantitative research. Measurement tools are intended to identify the important stressors among athletic directors and focus on managing them, whereas sport governing bodies use the tools to assess athletic directors’ current level of stress as a part of stress management and workplace health care initiatives.

Second, while the personal outcomes proposed in the current model are restricted to coaches, the coaching context also has the potential to influence athletes’ outcomes such as athlete burnout, satisfaction, commitment, effort, and turnover intention. Thus, once the proposed model is empirically confirmed by future research, the next step would be to incorporate relevant outcomes for individual athletes as a whole. 

Third, qualitative research should be conducted via an in-depth interview technique to reveal new findings that have not yet been explored and understand how athletic directors view their stress experience. Qualitative research involves the development of new concepts, the description of various real-world contexts, and the development of well-founded theories through observation, review of various records, and open interviews with a particular group of individuals [84]. The sport context has unique characteristics vis-à-vis other domains. Accordingly, the stress of individuals working in this environment may have unique characteristics. Thus, the first future research direction is to conduct qualitative research to understand the type and content of specific occupational stress in sport leadership.

Lastly, it is necessary to present a comprehensive management plan to reduce health impairment, improve the leadership effectiveness by understanding the status of occupational stress of athletic directors, and introduce any preventive programs. This requires research to support the importance of the prevention programs, accounting for social costs such as direct and indirect medical expenses and those related to the winning-at-all-cost mentality, abusive leadership style, and counterproductive behaviors caused by occupational stress. In addition, it is necessary to present a comprehensive policy direction for the prevention and management intervention of occupational stress.

## Figures and Tables

**Table 1:** Sources of occupational stress among sport leaders.

Job demand	Administration-related	Time pressure from the paperwork
		Amount of workloads
		Requirements for business techniques
		Budgetary pressure
		Public relation
		Policy compliance
	Competition-related	Pressure to win
		Role conflict
		Facility issues
Job autonomy	Unpredictable outcomes in the field	Level of opponents
		Characteristics of umpire
		Weather
	Uncontrollable athletic-related issues outside field	Player/coach turnover
		Injuries
		Deviant behaviors
Interpersonal conflict	Working with coaches and student-athletes	
	Working with parents and booster clubs	
Lack of rewards	Monetary rewards	
	Recognition	
	Opportunities for professional development	
Organizational culture	Male-oriented culture	

## Data Availability

No new data were created or analyzed in this study. Data sharing is not applicable to this article.

## References

[1] Hunter S.T., Tate B.W., Dzieweczynski J.L., Bedell-Avers K.E. (2011). Leaders make mistakes: A multilevel consideration of why. Leadersh. Q..

[2] Kaluza A.J., Boer D., Buengeler C., van Dick R. (2020). Leadership behaviour and leader self-reported well-being: A review, integration and meta-analytic examination. Work Stress.

[3] Campbell M., Baltes J.I., Martin A., Meddings K. (2007). The Stress of Leadership.

[4] Collins M.D., Jackson C.J. (2015). A process model of self-regulation and leadership: How attentional resource capacity and negative emotions influence constructive and destructive leadership. Leadersh. Q..

[5] Eubanks D.L., Mumford M.D. (2010). Leader errors and the influence on performance: An investigation of differing levels of impact. Leadersh. Q..

[6] Harms P., Credé M., Tynan M., Leon M., Jeung W. (2017). Leadership and stress: A meta-analytic review. Leadersh. Q..

[7] Martin J.J., Kelley B., Eklund R.C. (1999). A model of stress and burnout in male high school athletic directors. J. Sport Exerc. Psychol..

[8] Ryska T.A. (2002). Leadership styles and occupational stress among college athletic directors: The moderating effect of program goals. J. Psychol..

[9] Judge L.W., Judge I.L. (2009). Understanding the Occupational Stress of Interscholastic Athletic Directors. ICHPER-SD J. Res..

[10] Kaplan D. (2017). High School Athletic Directors Always Have Their Heads in the Games. New Jersey Monthly.

[11] Covell D., Walker S., Hess P., Siciliano J. (2012). Managing Sports Organizations.

[12] United Nations Inter-Agency Task Force on Sport for Development and Peace (2003). Sport for Development and Peace: Towards Achieving the Millennium Development Goals. United Nations.

[13] Driskell J.E., Salas E., Johnston J. (1999). Does stress lead to a loss of team perspective?. Group Dyn. Theory Res. Pract..

[14] Lee Y.H. (2020). The role of mindfulness and occupational stress in the goal orientations of development and winning. Sport Manag. Rev..

[15] Fletcher D., Scott M. (2010). Psychological stress in sports coaches: A review of concepts, research, and practice. J. Sports Sci..

[16] Olusoga P., Kenttä G. (2017). Desperate to quit: A narrative analysis of burnout and recovery in high-performance sports coaching. Sport Psychol..

[17] Caplan R., Cobb S., French J., Van Harrison R., Pinneau S. (1975). Demands and Worker Health: Main Effects and Organizational Differences.

[18] O’Connor D.B., Thayer J.F., Vedhara K. (2021). Stress and health: A review of psychobiological processes. Annu. Rev. Psychol..

[19] Le Fevre M., Matheny J., Kolt G.S. (2003). Eustress, distress, and interpretation in occupational stress. J. Manag. Psychol..

[20] Schneiderman N., Ironson G., Siegel S.D. (2005). Stress and health: Psychological, behavioral, and biological determinants. Annu. Rev. Clin. Psychol..

[21] NIOSH (1999). Stress at Work.

[22] Häusser J.A., Mojzisch A., Niesel M., Schulz-Hardt S. (2010). Ten years on: A review of recent research on the Job Demand–Control (-Support) model and psychological well-being. Work Stress.

[23] Hums M.A., MacLean J.C. (2017). Governance and Policy in Sport Organizations.

[24] Korn M. (2020). With Budgets Under Pressure, Colleges Cut Country-Club Staples Like Golf and Tennis. The Wall Stress Journal.

[25] Pedersen P.M., Thibault L. (2018). Contemporary Sport Management.

[26] Koehler M., Giebel N. (1997). Athletic Director’s Survival Guide.

[27] Pilar P.-M., Rafael M.-C., Félix Z.-O., Gabriel G.-V. (2019). Impact of sports mass media on the behavior and health of society. A systematic review. Int. J. Environ. Res. Public Health.

[28] O’Brien T. (2021). Know how to avoid Title IX violations when eliminating sports. Coll. Athl. Law.

[29] Lee Y.H., Chelladurai P., Kang C. (2018). Emotional labor in the dual role of teaching and coaching. Psychol. Rep..

[30] Richards K.A.R. (2015). Role socialization theory: The sociopolitical realities of teaching physical education. Eur. Phys. Educ. Rev..

[31] Fried G., Kastel M. (2020). Managing Sport Facilities.

[32] Karasek R.A. (1979). Job demands, job decision latitude, and mental strain: Implications for job redesign. Adm. Sci. Q..

[33] Bond F.W., Flaxman P.E., Bunce D. (2008). The influence of psychological flexibility on work redesign: Mediated moderation of a work reorganization intervention. J. Appl. Psychol..

[34] Shirom A., Nirel N., Vinokur A.D. (2006). Overload, autonomy, and burnout as predictors of physicians’ quality of care. J. Occup. Health Psychol..

[35] Lee Y.H., Cho H. (2021). The roles of different types of passion in emotional exhaustion and turnover intention among athletic coaches. Int. J. Sports Sci. Coach..

[36] Press A. (2017). Former Penn State Officials Gary Schultz, Tim Curley Begin Prison Terms. ESPN.

[37] Kerr Z.Y., Chandran A., Nedimyer A.K., Arakkal A., Pierpoint L.A., Zuckerman S.L. (2019). Concussion incidence and trends in 20 high school sports. Pediatrics.

[38] Jehn K.A., Mannix E.A. (2001). The dynamic nature of conflict: A longitudinal study of intragroup conflict and group performance. Acad. Manag. J..

[39] Lin W.-F., Lin Y.-C., Huang C.-L., Chen L.H. (2016). We can make it better: “We” moderates the relationship between a compromising style in interpersonal conflict and well-being. J. Happiness Stud..

[40] Fox S., Spector P.E., Miles D. (2001). Counterproductive work behavior (CWB) in response to job stressors and organizational justice: Some mediator and moderator tests for autonomy and emotions. J. Vocat. Behav..

[41] Jaramillo F., Mulki J.P., Boles J.S. (2011). Workplace stressors, job attitude, and job behaviors: Is interpersonal conflict the missing link?. J. Pers. Sell. Sales Manag..

[42] Kim S., Park S.M. (2014). Determinants of job satisfaction and turnover intentions of public employees: Evidence from US federal agencies. Int. Rev. Public Adm..

[43] Wakefield K.L. (2007). Team Sports Marketing.

[44] Peachey J.W., Zhou Y., Damon Z.J., Burton L.J. (2015). Forty years of leadership research in sport management: A review, synthesis, and conceptual framework. J. Sport Manag..

[45] Chelladurai P. (2014). Managing Organizations: For Sport and Physical Activity a Systems Perspective.

[46] Staurowsky E. (2014). College athletes’ rights in the age of the super conference: The case of the All Players United campaign. J. Intercoll. Sport.

[47] Todd J., Edwards J.R. (2020). Understanding parental support in elite sport: A phenomenological approach to exploring midget triple a hockey in the Canadian Maritimes. Sport Soc..

[48] Elliott S.K., Drummond M.J. (2017). Parents in youth sport: What happens after the game?. Sport Educ. Soc..

[49] Dorsch T.E., King M.Q., Tulane S., Osai K.V., Dunn C.R., Carlsen C.P. (2019). Parent education in youth sport: A community case study of parents, coaches, and administrators. J. Appl. Sport Psychol..

[50] Gould D., Lauer L., Rolo C., Jannes C., Pennisi N. (2006). Understanding the role parents play in tennis success: A national survey of junior tennis coaches. Br. J. Sports Med..

[51] Coakley J. (2016). Sports in Society: Issues and Controversies.

[52] Dextras-Gauthier J., Marchand A., Haines V. (2012). Organizational culture, work organization conditions, and mental health: A proposed integration. Int. J. Stress Manag..

[53] Burton L.J. (2015). Underrepresentation of women in sport leadership: A review of research. Sport Manag. Rev..

[54] Acosta R.V., Carpenter L.J. (2012). Women in Intercollegiate Sport: A Longitudinal, National Study. Thirty-Five Year Update, 1977–2012. Acosta-Carpent.

[55] Fagan K., Cyphers L. (2012). Five myths about Title IX. ESPN.

[56] Adriaenssens J., De Gucht V., Maes S. (2015). Causes and consequences of occupational stress in emergency nurses, a longitudinal study. J. Nurs. Manag..

[57] Bakker A.B., Demerouti E. (2007). The job demands-resources model: State of the art. J. Manag. Psychol..

[58] Halbesleben J.R., Buckley M.R. (2004). Burnout in organizational life. J. Manag..

[59] Halbesleben J.R.B., Leon M.R. (2014). Multilevel models of burnout: Separating group level and individual level effects in burnout research. Burnout at Work: A Psychological Perspective.

[60] Cohen S., Janicki-Deverts D., Miller G.E. (2007). Psychological stress and disease. JAMA.

[61] Melamed S., Shirom A., Toker S., Berliner S., Shapira I. (2006). Burnout and risk of cardiovascular disease: Evidence, possible causal paths, and promising research directions. Psychol. Bull..

[62] Fortes A., Tian L., Huebner S. (2020). Occupational stress and employees complete mental health: A cross-cultural empirical study. Int. J. Environ. Res. Public Health.

[63] Griffeth R.W., Hom P.W., Gaertner S. (2000). A meta-analysis of antecedents and correlates of employee turnover: Update, moderator tests, and research implications for the next millennium. J. Manag..

[64] Murphy L.R., DuBois D., Hurrell J.J. (1986). Accident reduction through stress management. J. Bus. Psychol..

[65] Frone M.R. (2008). Are work stressors related to employee substance use? The importance of temporal context assessments of alcohol and illicit drug use. J. Appl. Psychol..

[66] García-Izquierdo M., Meseguer de Pedro M., Ríos-Risquez M.I., Sánchez M.I.S. (2018). Resilience as a moderator of psychological health in situations of chronic stress (burnout) in a sample of hospital nurses. J. Nurs. Scholarsh..

[67] Hengartner M.P., van der Linden D., Bohleber L., von Wyl A. (2017). Big five personality traits and the general factor of personality as moderators of stress and coping reactions following an emergency alarm on a Swiss University Campus. Stress Health.

[68] Vallerand R.J., Blanchard C., Mageau G.A., Koestner R., Ratelle C., Léonard M., Gagné M., Marsolais J. (2003). Les passions de l’ame: On obsessive and harmonious passion. J. Personal. Soc. Psychol..

[69] Lucidi F., Pica G., Mallia L., Castrucci E., Manganelli S., Bélanger J., Pierro A. (2016). Running away from stress: How regulatory modes prospectively affect athletes’ stress through passion. Scand. J. Med. Sci. Sports.

[70] Lafrenière M.-A.K., Jowett S., Vallerand R.J., Carbonneau N. (2011). Passion for coaching and the quality of the coach–athlete relationship: The mediating role of coaching behaviors. Psychol. Sport Exerc..

[71] Swanson S., Kent A. (2017). Passion and pride in professional sports: Investigating the role of workplace emotion. Sport Manag. Rev..

[72] Dewe P., Cox T., Ferguson E. (1993). Individual strategies for coping with stress at work: A review. Work Stress.

[73] Grandey A.A. (2000). Emotional regulation in the workplace: A new way to conceptualize emotional labor. J. Occup. Health Psychol..

[74] Gan Y., Hu Y., Zhang Y. (2010). Proactive and preventive coping in adjustment to college. Psychol. Rec..

[75] Lee Y., Chelladurai P. (2018). Emotional intelligence, emotional labor, coach burnout, job satisfaction, and turnover intention in sport leadership. Eur. Sport Manag. Q..

[76] Uchino B.N. (2009). Understanding the links between social support and physical health: A life-span perspective with emphasis on the separability of perceived and received support. Perspect. Psychol. Sci..

[77] Pearlin L.I., Menaghan E.G., Lieberman M.A., Mullan J.T. (1981). The stress process. J. Health Soc. Behav..

[78] Eisenberger R., Stinglhamber F. (2011). Perceived Organizational Support: Fostering Enthusiastic and Productive Employees.

[79] Cropanzano R., Greenberg J. (1997). Progress in organizational justice: Tunneling through the maze. Int. Rev. Ind. Organ. Psychol..

[80] Bies R.J. (1986). Interactional justice: Communication criteria of fairness. Res. Negot. Organ..

[81] Aryee S., Chen Z.X., Sun L.-Y., Debrah Y.A. (2007). Antecedents and outcomes of abusive supervision: Test of a trickle-down model. J. Appl. Psychol..

[82] Cohen-Charash Y., Spector P. (2012). The role of justice in organizations: A meta-analysis. Organ. Behav. Hum. Decis. Processes.

[83] Roy J., Bastounis M., Minibas-Poussard J. (2012). Interactional justice and counterproductive work behaviors. The mediating role of negative emotion. Soc. Behav. Personal. Int. J..

[84] Creswell J.W., Poth C.N. (2016). Qualitative Inquiry and Research Design: Choosing among Five Approaches.

